# Mandatory Vaccination Against COVID-19: Twitter Poll Analysis on Public Health Opinion

**DOI:** 10.2196/35754

**Published:** 2022-06-21

**Authors:** Valentin Ritschl, Fabian Eibensteiner, Erika Mosor, Maisa Omara, Lisa Sperl, Faisal A Nawaz, Chandragiri Siva Sai, Merisa Cenanovic, Hari Prasad Devkota, Mojca Hribersek, Ronita De, Elisabeth Klager, Eva Schaden, Maria Kletecka-Pulker, Sabine Völkl-Kernstock, Harald Willschke, Christoph Aufricht, Atanas G Atanasov, Tanja Stamm

**Affiliations:** 1 Section for Outcomes Research Medical University of Vienna Vienna Austria; 2 Ludwig Boltzmann Institute for Arthritis and Rehabilitation Vienna Austria; 3 Division of Pediatric Nephrology and Gastroenterology, Department of Pediatrics and Adolescent Medicine, Comprehensive Center for Pediatrics Medical University of Vienna Vienna Austria; 4 Ludwig Boltzmann Institute for Digital Health and Patient Safety Medical University of Vienna Vienna Austria; 5 College of Medicine Mohammed Bin Rashid University of Medicine and Health Sciences Dubai United Arab Emirates; 6 Amity Institute of Pharmacy Amity University Uttar Pradesh India; 7 Sarajevo Bosnia and Herzegovina; 8 Graduate School of Pharmaceutical Sciences Kumamoto University Kumamoto Japan; 9 Program for Leading Graduate Schools Kumamoto University Kumamoto Japan; 10 Indian Council of Medical Research-National Institute of Cholera and Enteric Diseases West Bengal India; 11 Department of Anaesthesia, Intensive Care Medicine and Pain Medicine Medical University of Vienna Vienna Austria; 12 Institute for Ethics and Law in Medicine University of Vienna Vienna Austria; 13 Department of Child and Adolescent Psychiatry Medical University of Vienna Vienna Austria; 14 Institute of Genetics and Animal Biotechnology of the Polish Academy of Sciences Jastrzebiec Poland

**Keywords:** COVID-19, SARS-CoV-2, vaccine, vaccination, Twitter, survey, mandatory vaccination, vaccination hesitancy, coronavirus, hesitancy, social media, questionnaire, mandatory, support, poll, opinion, public health, perception

## Abstract

**Background:**

On January 30, 2020, the World Health Organization Emergency Committee declared the rapid worldwide spread of COVID-19 a global health emergency. By December 2020, the safety and efficacy of the first COVID-19 vaccines had been demonstrated. However, international vaccination coverage rates have remained below expectations (in Europe at the time of manuscript submission). Controversial mandatory vaccination is currently being discussed and has already been introduced in some countries (Austria, Greece, and Italy). We used the Twitter survey system as a viable method to quickly and comprehensively gather international public health insights on mandatory vaccination against COVID-19.

**Objective:**

The purpose of this study was to better understand the public’s perception of mandatory COVID-19 vaccination in real time using Twitter polls.

**Methods:**

Two Twitter polls were developed (in the English language) to seek the public’s opinion on the possibility of mandatory vaccination. The polls were pinned to the Digital Health and Patient Safety Platform’s (based in Vienna, Austria) Twitter timeline for 1 week in mid-November 2021, 3 days after the official public announcement of mandatory COVID-19 vaccination in Austria. Twitter users were asked to participate and retweet the polls to reach the largest possible audience.

**Results:**

Our Twitter polls revealed two extremes on the topic of mandatory vaccination against COVID-19. Almost half of the 2545 respondents (n=1246, 49%) favor mandatory vaccination, at least in certain areas. This attitude contrasts with the 45.7% (n=1162) who categorically reject mandatory vaccination. Over one-quarter (n=621, 26.3%) of participating Twitter users said they would never get vaccinated, as reflected by the current Western European and North American vaccination coverage rate. Concatenating interpretation of these two polls should be done cautiously as participating populations might substantially differ.

**Conclusions:**

Mandatory vaccination against COVID-19 (in at least certain areas) is favored by less than 50%, whereas it is opposed by almost half of the surveyed Twitter users. Since (social) media strongly influences public perceptions and views, and social media discussions and surveys are specifically susceptible to the “echo chamber effect,” the results should be interpreted as a momentary snapshot. Therefore, the results of this study need to be complemented by long-term surveys to maintain their validity.

## Introduction

Many mitigation measurements, mostly nonpharmaceutical interventions, have been undertaken on local, national, and international levels to reduce the transmission of COVID-19 since the beginning of this pandemic [1-6]. As Sridhar and Gurdasani [7] discussed in January 2021, immunity can be boosted safely through vaccination in many infectious diseases, while achievement of herd immunity through SARS-CoV-2 infection is not a strategy worth considering due to little guarantee of success while putting a high toll on morbidity and mortality. Therefore, the return to prepandemic normality may rely on the success of vaccine-induced immunity to prevent severe disease and limit dissemination [8]. Although the first COVID-19 vaccines were quickly proven safe and efficacious, and were approved by regulatory authorities in December 2020 [9-12], global vaccination coverage has not been achieved for several reasons. Vaccination coverage rates largely depend on a country’s wealth and other factors that influence the vaccination behavior of a country’s citizens. As a result, SARS-CoV-2 variants continue to emerge, triggering disease episodes and slowing or even reversing the reopening of societies and economies [13].

Widespread public acceptance of vaccines continues to be a challenging endeavor requiring accountancy of complex socioeconomic factors on the level of international policy makers, national and local public health officials, and professional and community organizations [14]. A large study from four metropolitan areas of the United States found more than 20% of participants reluctant to vaccinate. Participants expressed concerns on efficacy and safety while also questioning the severity of a COVID-19 infection [15]. In a Canadian study, participants who did not plan to get vaccinated were also less likely to retain mitigation measures such as wearing face masks and practicing physical distancing [16].

Outreach to the public providing necessary information regarding COVID-19 has been achieved over several communication channels, such as traditional media and different social media platforms [17]. Public health implications of social media platforms such as Twitter have been studied before and with increasing intensity during the COVID-19 pandemic. Examples include public perception of antibiotic use and misuse, human papillomavirus vaccination on Twitter and analysis of boosted vaccination hesitancy, and the re-emergence of measles in the United States after its elimination [18-20]. During the COVID-19 pandemic, substantial effort has also been drawn to study symptoms for COVID-19 screening, dissemination of medical information and misinformation, the emergence of conspiracy theories, and discussions and emotions associated with COVID-19 on Twitter [21-25]. Longitudinal sentiment analysis of Twitter discussions around COVID-19 revealed a peak of percentages of tweets expressing fear in mid-March 2020 after the initial declaration of the pandemic, with the lowest point in early November 2020 when the first COVID-19 vaccines were announced. With the increasing perspective of promising vaccine results, the percentage of tweets expressing trust increased while fear declined [26]. With Twitter hosting about 353 million monthly active users and incorporating an inbuilt anonymous polling tool, it allows for potential insights into pressing public health topics on an international level with real-time feedback [27,28]. In a previous Twitter poll study on the public’s perceptions of the currently available COVID-19 vaccines, Eibensteiner et al [29] detected a high willingness to get vaccinated despite high levels of uncertainty regarding the available vaccine’s safety in February 2021. A later published study analyzing 4 million tweets since the beginning of the pandemic added that Twitter bots or political activists partly generated vaccine opposition content.

In contrast, positive content on COVID-19 vaccination was produced mainly by well-known individuals and organizations [30]. A recent study by Germani and Biller-Andorno [31] also shows that those against vaccination increasingly participate in discussions on Twitter and disseminate their content from a pool of strong influencers such as political activists, authors, or artists. Donald Trump, a previous president of the United States, was the most influential disseminator of antivaccination content on Twitter (before his account was suspended). At this point, it should be emphasized that, of course, not only social media such as Twitter but also other factors can lead to vaccination hesitancy. Truong et al [32] mentioned in their review, for example, demographic factors (ethnicity, age, gender, pregnancy, education, and employment), personal responsibility and risk perception, trust in health authorities, and the (perceived) safety and efficacy of a new vaccine, as well as a lack of information or incorrect information about vaccines.

Mandatory vaccination against COVID-19 for the public or health care workers has been a recent focus of attention in many European countries, including Austria, Germany, and the United Kingdom [33-35]. In Austria (general public 18 years or older) and Germany (employees in hospitals and care facilities), mandatory vaccination is scheduled for spring 2022. Recently, Italy approved mandated vaccines for everyone over 50 years of age and Greece for people older than 60 years [34-36]. A survey in Germany on 20,000 households in early summer 2020 revealed that about 50% of Germany’s residents would favor mandatory vaccination [37].

With the recent announcement of mandatory COVID-19 vaccination in some western democracies (eg, Austria or Germany), our study aims to survey the public’s attitude on this matter. As has already been shown before, the fast-paced dynamic of this pandemic requires online survey tools to gain immediate large-scale international public health insights [23,26,29,38]. Therefore, we used the Twitter polling tool to rapidly collect and analyze the public’s opinion on mandatory COVID-19 vaccination to understand endorsement and refusal, possibly aiding policy makers on this highly relevant and timely topic. This study aimed to better understand public perceptions of mandatory COVID-19 vaccination in real time using Twitter polls.

## Methods

### Overview

To meet the objective of this study and better understand public perceptions of mandatory COVID-19 vaccination, we conducted two Twitter polls. For this purpose, we used the Twitter account of the Digital Health and Patient Safety Platform (DHPSP; Twitter handle @DHPSP) [39]. The DHPSP was founded by the Ludwig Boltzmann Institute for Digital Health and Patient Safety, established in Austria in 2019 [29].

**Figure 1:**
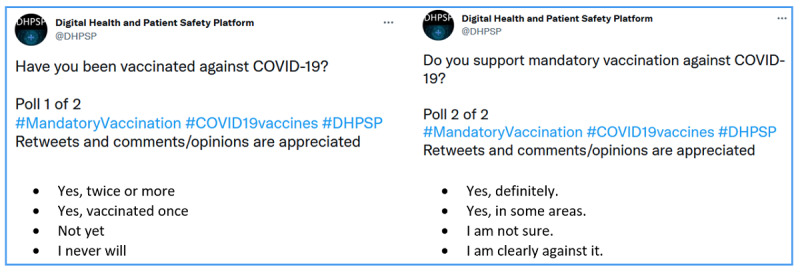
Structure of the two Twitter polls. DHPSP: Digital Health and Patient Safety Platform.

For this study, we distributed two Twitter polls online via the Twitter account @DHPSP between November 22 and 29, 2021. The polls were developed within the project expert team and evaluated in multiple rounds to ensure the best possible readability and comprehensibility. Poll 1 addressed whether participants had already been vaccinated (“Have you been vaccinated against COVID-19?”), whereas poll 2 asked about opinions about a possible mandatory vaccination for COVID-19 (“Do you support mandatory vaccination against COVID-19?”). Both polls were linked (poll 2 was posted as a comment under poll 1) and pinned to the top of the DHPSP Twitter timeline during the poll period. Pinning a tweet permanently places it at the top of a Twitter user’s account so that any new visitors will see this tweet at the top of the visited user’s timeline. The poll questions, including relevant hashtags for categorization, are limited to 280 characters on Twitter. Twitter allows up to four responses with a limit of 25 characters including spaces for each poll. Therefore, both polls had four responses ranging from complete agreement (“Yes, twice or more” and “Yes, definitely”) to complete disagreement (“I never will” and “I am clearly against it”) in the manner of a four-point response scale. Both surveys were categorized with the following hashtags to increase visibility and facilitate analysis: #MandatoryVaccination, #COVID19vaccines, and #DHPSP. Figure 1 shows the detailed structure of the two polls as they were distributed on Twitter.

**Figure 2:**
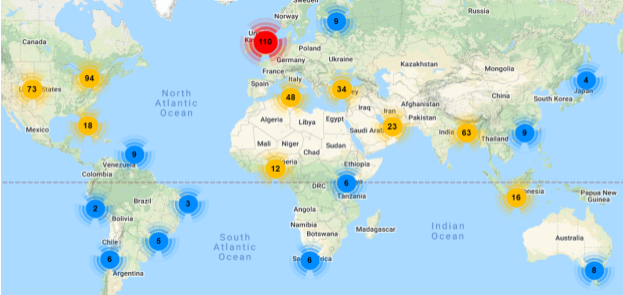
Main locations of the Digital Health and Patient Safety Platform's (DHPSP) Twitter followers (note: these data cover only the fraction of the DHPSP's followers who indicated their location in their account information on Twitter).

Once the polls were launched, the first accounts to see them in their Twitter timelines were DHPSP Twitter followers. Twitter polls are anonymous and do not allow respondents’ characteristics (eg, gender) to be assessed. Therefore, to obtain the characteristics of the audience that were first exposed to the polls, we attempted to analyze the characteristics of @DHPSP’s followers via the online tool Followerwonk [40] on December 1, 2021. In total, the Twitter account @DHPSP had 943 followers at the time of the analysis. Of these, 206 (21.8%) were male, 133 (14.1%) were female, and 604 (64.1%) did not indicate their gender on Twitter. Overall, 137 (14.5%) DHPSP followers had more than 5000 followers on their own, 320 (33.9%) had between 500 and 5000 followers, and 486 (51.5%) had less than 500 followers. The geographical distribution of the @DHPSP follower network can be seen in Figure 2. In addition, the DHPSP network includes 225 people on the mailing list, 306 people on LinkedIn, and 1757 Facebook followers.

The text of the polls asked for retweets and discussion (“Retweets and comments/opinions are appreciated”; Figure 1), and with each new retweet, the polls gained an additional audience (consisting of the followers of the retweeting accounts). To gain extra visibility, members and subscribers to the DHPSP platform email list [39] were asked to support the polls by voting, retweeting, and sharing them through various additional networking approaches via emails or direct messages on social media. Various social media accounts of DHPSP members were also used to post hyperlinks to the Twitter polls. In addition, information about the polls was disseminated through DHPSP’s Facebook [41] and LinkedIn accounts [42].

To characterize the user population that retweeted the polls under study, we conducted a hashtag analysis using the Symplur Signals online tool [43]. We analyzed (in terms of the number of retweets, users, locations, and languages) all tweets that contained the unique combination of the hashtags #MandatoryVaccination, #COVID19vaccines, and #DHPSP at the end of the poll period on November 29, 2021. To ensure accuracy and limit interference from other Twitter discussions on this topic, a Twitter search was conducted before the start of the surveys on November 21, 2021, which confirmed that this hashtag combination had never been used before. Symplur Signals was also used for sentiment analysis of all tweets containing this unique combination of hashtags. For additional analysis of the gender and age distribution of direct retweets, the tool Tweepsmap was applied using its “Tweet Reach” feature (quote tweets not included due to the specifics of the used tool).

### Ethical Considerations

No ethical approval was required for this study as it does not fall within the scope of the Austrian Medical Ethics Act. Individual votes, retweets, and comments of any kind were anonymized using Symplur Signals. All data presented in this manuscript are anonymous. Thus, the data collected do not fall within the scope of the General Data Protection Regulation [44]. As follow-up information, the voting counts are immediately revealed to users as soon as they vote through their Twitter account. The parameters analyzed beyond the survey results, such as the number of followers and retweets, are based on online publicly available data.

## Results

**Figure 3:**
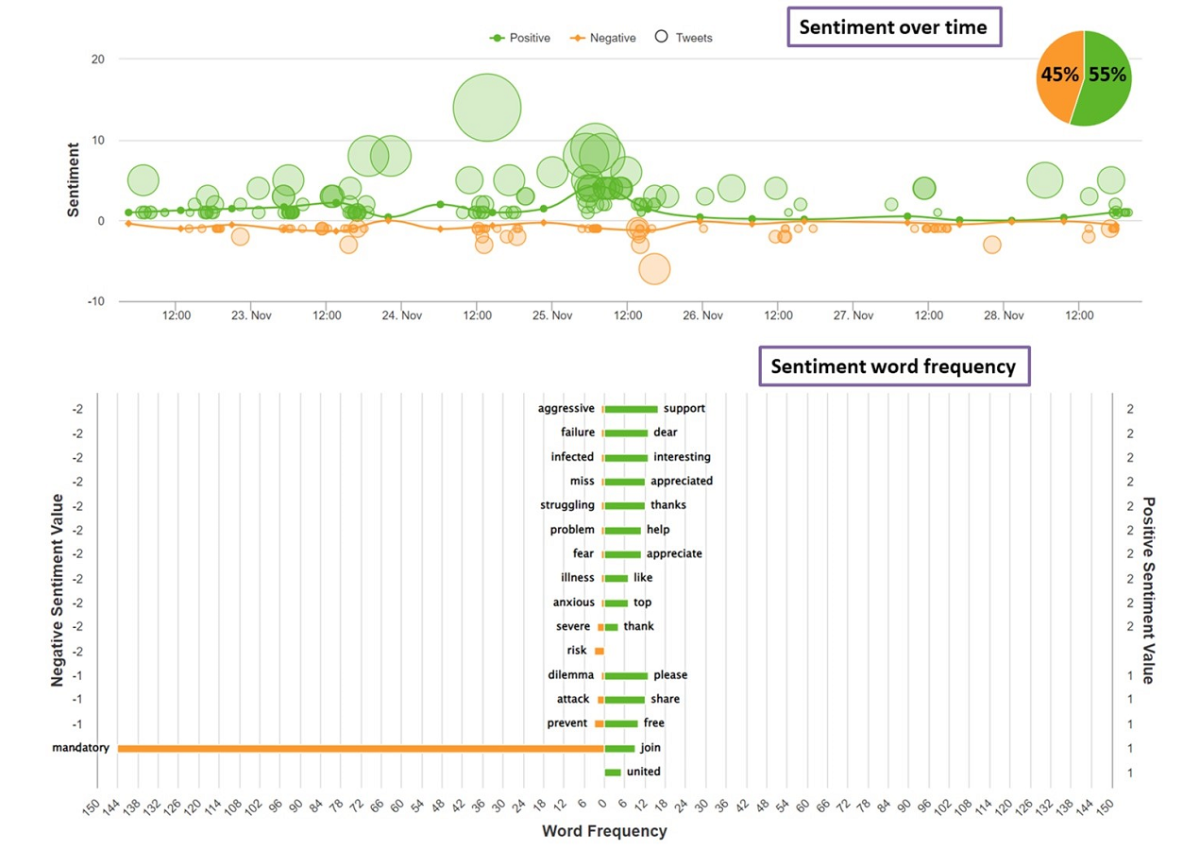
Sentiment analysis of the poll's retweets containing the unique combination of the following hashtags: #MandatoryVaccination, #COVID19vaccines, and #DHPSP (Digital Health and Patient Safety Platform). The upper panel indicates sentiment over time and the overall sentiment scores (45% negative and 55% positive), and the lower panel displays sentiment word frequencies.

Both Twitter polls were pinned to the timeline of the DHPSP’s Twitter account (Twitter handle @DHPSP; Table 1) for 7 days, beginning on November 22, 2021. The gender and age distribution of the Twitter accounts that directly retweeted both polls (poll 1, n=178; poll 2, n=189) are depicted in Table 2. Sentiment analysis of all tweets featuring the polls demonstrated 45% (32 primary tweets with a sentiment score range from –0.5894 to –0.062) negative sentiment and 55% (50 primary tweets with a sentiment score range from 0.9338 to 0.7536) positive sentiment of the analyzed tweets (Figure 3).

Poll 1 (“Have you been vaccinated against COVID-19?”) received a total of 2365 votes (199,902 views), whereas poll 2 (“Do you support mandatory vaccination against COVID-19?”) received a total of 2545 votes (200,939 views). Upon analysis of the polls’ retweets that contained the unique combination of the hashtags #MandatoryVaccination, #COVID19vaccines, and #DHPSP, a total of 2073 tweets from 442 users (one retweet: n=272, 61.5%; two retweets: n=100 users, 22.6%; three or more retweets: n=70, 15.8%) were identified. The polls, including all retweets, summed up to a total of 32,594,283 views on Twitter. The top locations of Twitter users retweeting the polls were the United States (n=59, 6.3%), Canada (n=41, 4.5%), and the United Kingdom (n=17, 1.5%). However, most of the users did not indicate their location. A summary of these details is given in Table 3.

Of the Twitter users who responded to poll 1 (“Have you been vaccinated against COVID-19?”), 63.4% (1499/2365) agreed with “Yes, twice or more”; therefore, almost two-thirds of users who answered this question reported to be fully vaccinated. More than one-quarter (621/2365, 26.3%) of Twitter users expressed that they will never get vaccinated against COVID-19 (they voted “I never will”). Together, these two groups represent the extremes regarding vaccination and represent 89.6% (2120/2365) of all answers to this question. Of the 2365 respondents, 111 (4.7%) reported that they have not yet been vaccinated but do not rule out the possibility of getting vaccinated (“Not yet.”). The remaining 5.7% (n=134) of Twitter users had been vaccinated but have not yet received a second vaccine dose (“Yes, vaccinated once”).

**Figure 4:**
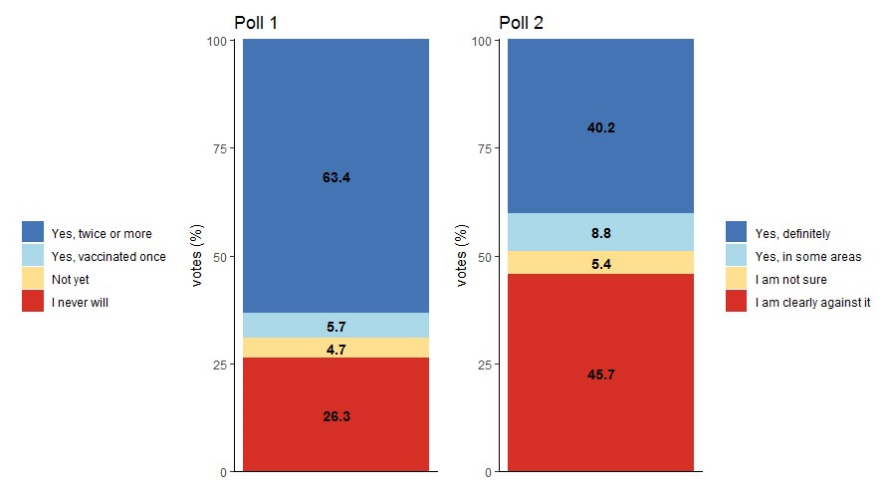
Twitter users' answers to poll 1 ("Have you been vaccinated against COVID-19?"; respondents n=2365) and poll 2 ("Do you support mandatory vaccination against COVID-19?"; respondents n=2545).

Poll 2 examined Twitter users’ attitudes toward possible mandatory vaccination (“Do you support mandatory vaccination against COVID-19?”). In this poll, 40.2% (1022/2545) of all respondents indicated (“Yes, definitely”) that they would support mandatory vaccination against COVID-19. In addition, 8.8% (224/2545) indicated that they would support mandatory vaccination in certain areas (eg, for certain professions; “Yes, in some areas”). In contrast to the vaccine supporters, almost half of the respondents (1162/2545, 45.7%) are strictly against mandatory vaccination (“I am clearly against it”). A small percentage of 5.4% (137/2545) of Twitter users stated that they do not yet have an opinion on mandatory vaccination against COVID-19 (“I am not sure”). A detailed summary of the responses to both polls is given in Figure 4.

**Table 1:** Digital Health and Patient Safety Platform’s Twitter follower characteristics.

		Followers (n=943), n (%)
**Gender**		
	Male	206 (21.8)
	Female	133 (14.1)
	Not stated	604 (64.1)
**Follower counts**		
	<500	486 (51.5)
	500-5000	320 (33.9)
	>5000	137 (14.5)
**Account ages (years)**		
	<1	86 (9.1)
	1-5	325 (34.5)
	>5	532 (56.4)
**Languages**		
	English	586 (62.1)
	Spanish	46 (4.9)
	Other	301 (31.9)

**Table 2:** Gender and age distribution of Twitter accounts that retweeted the polls.

			Twitter accounts, n (%)
**Poll 1 (n=178)**			
	**Distribution by gender poll 1: direct retweets**		
		Male	102 (57.3)
		Female	61 (34.4)
		Businesses/groups	15 (8.3)
	**Distribution by age poll 1: direct retweets**		
		10-23 years	20 (11.4)
		24-64 years	142 (80.0)
		≥65 years	16 (8.6)
**Poll 2 (n=189)**			
	**Distribution by gender poll 2: direct retweets**		
		Male	112 (59.6)
		Female	69 (36.4)
		Businesses/groups	8 (4)
	**Distribution by age poll 2: direct retweets**		
		10-23 years	28 (14.8)
		24-64 years	154 (81.5)
		≥65 years	7 (3.7)

**Table 3:** Analysis of the poll’s retweets containing the unique combination of the following hashtags: #MandatoryVaccination, #COVID19vaccines, and #DHPSP (Digital Health and Patient Safety Platform).

		Twitter users (n=943), n (%)
**Top locations of Twitter users^a^**		229 (24.3)
	United States	59 (6.3)
	Canada	41 (4.5)
	United Kingdom	17 (1.5)
**Number of users that retweeted^b^**		442 (46.9)
	One retweet	272 (61.5)
	Two retweets^c^	100 (22.6)
	Three or more retweets^c^	70 (15.8)
**Top languages^d^**		
	English	1969 (95.0)
	Other languages	104 (5.0)

^a^Determined based on data derived just from the users who indicated their location in their account information on Twitter. While interpreting the data, the readers should be aware that 75.7% of the 943 users did not provide location information on their profiles.

^b^The total number of retweets was 2073.

^c^Including “regular” retweets, retweets with comments, and “quote retweets” (whereby a hyperlink to the original tweet is inserted in the newly composed tweet).

^d^The most used languages are indicated. All other tweet languages accounted for less than 0.5% each.

## Discussion

Our Twitter polls showed two extremes on the issue of compulsory vaccination against COVID-19. Almost half of the respondents favor compulsory vaccination, at least for certain professional groups. However, this is in contrast to nearly as many people who categorically reject compulsory vaccination, indicating that a proportion of vaccinated people voted against mandating vaccination. In line with recent works building the methodological basis and outlining the possible benefits of Twitter polling to gain quick insights into the public’s attitudes on timely matters [28,29], we aimed to explore the public’s opinion on mandatory COVID-19 vaccination by using Twitter polls. This topic is currently of high interest as recently mandatory COVID-19 vaccination has been scheduled for spring 2022 in Austria and Germany [34,35]. This timely and important Twitter survey reveals that mandatory vaccination against COVID-19 (in at least certain areas) is supported by less than 50%, whereas it is opposed by almost half of the surveyed Twitter users.

We used an established Twitter network (the DHPSP’s Twitter account) to pin our Twitter polls to generate high outreach and a high number of respondents in this study. This work complements our initial Twitter survey on the perceived safety of the available COVID-19 vaccines and participants’ confidence or hesitancy to get vaccinated [29]. In February 2021, 83% of Twitter users participating in polls posted back then stated that they would definitely get vaccinated against COVID-19. About 70% of participants indicated that they received at least their first dose in the current poll. The percentage of participants expressing their reluctance to get vaccinated increased from 8% to 26%. This data is in line with the current vaccination rates in Western European countries (United Kingdom, Germany, Austria, France, Spain, and Italy) and the United States, with 71% to 83% of people being at least partly vaccinated [45], and above the world average of 55% [45]. A direct comparison of these two Twitter polls can be carefully made, considering that the initial Twitter network was the same, even though participating users might substantially differ. Interpretability is further limited by the DHPSP’s follower base consisting mainly of younger educated individuals with scientific backgrounds, mainly due to the selective science-based content that attracted such followers and their interest in science. This concern is in line with several studies associating COVID-19 vaccine hesitancy with lower educational levels [46-49]. Comparability of other studies with our study is not straightforward, as the logic and infrastructure of Twitter surveys make the retrospective characterization of the sample difficult. Available information about Twitter users is limited (eg, in terms of location and language). Despite the composition of the original sample of “younger, educated people,” we reached a total of 26.3% (621/2365) of convinced nonvaxxers in our survey, which is in line with the results reported in other studies, such as the one by Hacquin and colleagues [49]. We explain this phenomenon by the fact that while the original sample was indeed enriched with younger, (better formally) educated individuals, each new retweet immediately adds the followers of each new retweeting account as an audience for the surveys. In this way, we seem to have achieved an equilibrium consistent with opinion patterns among Twitter users, which may explain why the proportion of nonvaxxers in our study was similar to that observed in previous work.

Our findings agree with the previously reported percentage of 26% of participants stating that they will never get vaccinated against COVID-19, with about 5% remaining undecided. In the current literature, COVID-19 vaccine hesitancy is further evident among university students (14%), medical students (23%), and health care workers (28%) [50-52]. A large-scale survey in Ireland and the United Kingdom segmented the previously published high percentages of COVID-19 vaccination hesitancy/refusal (eg, 33% in the United States) to 25% to 26% vaccination hesitancy and 6% to 9% resistance [53]. Although we aimed to formulate our poll questions and answers clearly and discriminatively (eg, “I never will”), further interpretation of these results due to missing demographics and other indicators of socioeconomic status, health literacy, and political and religious views was not possible. In addition, the study published by Murphy et al [53] was conducted in Spring 2020, at a time when no COVID-19 vaccines were yet available. Vaccination refusal may not only affect the course of this pandemic but has also been the focus of discussion among the prevention of other vaccine-preventable and potentially deadly diseases such as measles [54].

Our Twitter polls revealed high rates of being vaccinated against COVID-19 at least once, while 40% of participants supported mandatory vaccination for the public. Such concatenating interpretation of these two polls needs to be done cautiously, as the participating populations might substantially differ between both polls. Mandatory vaccination against COVID-19 is currently under rigorous discussion in many European countries and among health and home care workers [33,55,56]. Austria is currently the first western democracy, followed by Germany, to officially announce mandatory vaccination against COVID-19 for the public [34,35]. This would not be the first time mandatory vaccination has been enforced. For example, in England and Imperial Germany, between 1874 and 1975, vaccination against smallpox was compulsory, resulting in substantially reduced mortality rates [57]. However, ethical, medical, and philosophical reasons supporting and opposing mandatory vaccination involve complex socioeconomic and psychological perspectives [58-60] that are out of this paper’s scope and will not be further discussed.

Several studies have assessed the public’s opinion on mandatory COVID-19 vaccination in German, French, Greek, Austrian, American, Pakistani, and Italian individuals. A broad range favored mandatory COVID-19 vaccination from 17% to 74% of survey participants. Five of these studies were conducted before COVID-19 vaccines were available and before the official public announcement of mandatory vaccination against COVID-19 for the public in Austria [37,61-66].

Therefore, this is the first study analyzing the public’s attitude on mandatory COVID-19 vaccination immediately following (3 days) the official public announcement of the Austrian government, which is the first western democracy to mandate vaccination for its entire population [34]. This timely analysis on an international scale provides valuable insights into changes in the public’s attitude and general beliefs toward COVID-19 vaccine mandates that may aid policy makers in strategizing similar paths to Austria.

The strengths of this study lay in the rapid and timely assessment on an international scale with clear and concise information on the publics’ attitude. As previously discussed in our prequel study, restrictions of word counts in Twitter polling might serve as a strength for concise and well-formulated surveys, possibly aiding a higher number of respondents in comparison to traditional surveys. On the other hand, it substantially limits the interpretability of results, as no additional information may be retrieved, and the formulation of more complex questions and clarification statements is hampered [29]. For example, it could be that the wording of the second answer of the second poll, “yes, in some areas” I am in favor of compulsory vaccination, may be understood by some respondents as “region,” “district,” or another geographical unit. In the original conception, however, the term “area” was meant rather as “branch” or “profession.” A misunderstanding cannot be ruled out and must be considered when interpreting the results. Distribution of the Twitter polls via a pre-existing network might also influence sample selection and cause bias further challenged by a lack of baseline characteristics of the survey participants. This is potentiated by the “echo chamber effect,” exposing social media users to curated content most likely aligning with their pre-existing beliefs based on their previous social media behavior [67,68]. This also includes the previous observation that individuals exposed to negative opinions on human papillomavirus vaccination were more likely to share these on Twitter, in contrast to those exposed to neutral or positive opinions [69]. An explicit limitation also lies in the extremely limited concatenating interpretation of the different Twitter polls, as the participating user populations might substantially differ between the two polls, and comparability cannot be achieved due to the polls’ anonymity. Thus, a direct transferability of the results to individual countries is impossible or only possible to a limited extent. In addition, poll manipulation by exploiting multiple users due to the polls’ anonymity needs to be kept in mind upon interpreting these results, as already mentioned by Vidal-Alaball et al [28]. We used Symplur Signals for sentiment analysis of all tweets. This automated text-mining tool helped us get an impression of the sentiment. Still, it has to be mentioned that this is not equal to classical qualitative data analysis.

When interpreting the data, it is important to remember that the two surveys involved self-selected users of a popular social media platform who self-reported their COVID-19 vaccination status and their opinion on introducing such mandatory vaccination. Objective data, for example, on vaccination status, could not be collected in this study.

In summary, mandatory vaccination against COVID-19 is supported by less than 50% of Twitter users and opposed by almost half of the Twitter users surveyed in this study. Refusal rates of COVID-19 vaccination are prevalent among 26% of surveyed Twitter users. These findings are reflected by the current vaccination coverage rates and align with the existing literature. Public perceptions and views on health issues are heavily influenced by social media, being specifically susceptible to the “echo chamber effect,” underscoring the importance of using social media surveys to understand the public’s views on health in real time to inform public health messages and communications efforts.

## Abbreviations

DHPSP: Digital Health and Patient Safety Platform

## Data Availability

Raw data are available upon reasonable request.
